# Tumor Suppressive Maspin-Sensitized Prostate Cancer to Drug Treatment Through Negative Regulating Androgen Receptor Expression

**DOI:** 10.3389/fcell.2020.573820

**Published:** 2020-10-26

**Authors:** Sijie Tang, Xueqi Lian, Jiajia Jiang, Huiying Cheng, Jiaqian Guo, Can Huang, Hong Meng, Xiaohua Li

**Affiliations:** ^1^The AoYang Cancer Institute, Jiangsu University, Suzhou, China; ^2^Perinatology Research Branch, Eunice Kennedy Shriver National Institute of Child Health and Human Development, National Institutes of Health, Detroit, MI, United States; ^3^The Laboratory of Clinical Genomics, Hefei KingMed Diagnostics Laboratory, Hefei, China; ^4^National Center for Gene Testing Technology Application & Demonstration (Anhui), Hefei, China

**Keywords:** maspin, androgen receptor, HDAC inhibitor, chemotherapy, castration-resistant prostate cancer

## Abstract

Overactivation of androgen receptor (AR)-mediated signal has been extensively implicated in prostate cancer (CaP) development, progression, and recurrence, which makes it an attractive therapeutic target. Meanwhile, as an endogenous inhibitor of histone deacetylase 1 (HDAC 1), tumor-suppressive mammary serine protease inhibitor (maspin) was reported to sensitize drug-induced apoptosis with a better therapeutic outcome in CaP, but the relationship between AR and maspin remains unclear. In the current study, treatment of 5′-Aza or MS-275/enzalutamide induced poly (ADP-ribose) polymerase (PARP) cleavage and p-H2A.X in CaP cells with an increase of maspin expression but a decrease of AR. Then, treatment with protease inhibitor MG132 did not rescue the above drug-induced loss of AR. In addition, modulation of maspin expression by gene recombinant or siRNA technology showed an inverse correlation between expression of maspin and AR, consequently affecting the AR-regulated downstream gene transcription (e.g., NKX3.1 and TMPRSS2). Bioinformatics analysis of the data extracted from the National Center for Biotechnology Information Gene Expression Omnibus (NCBI GEO) database also revealed an inverse correlation between low maspin expression and high AR level in advanced CaP. Furthermore, chromatin immunoprecipitation (ChIP) assay using anti-maspin antibody identified that a portion of AR promoter sequence was co-precipitated and presented in the immunoprecipitated complex. Finally, maspin-mediated repression of AR was induced by treatment of MS-275, which promoted enzalutamide treatment efficacy with decrease of prostate-specific antigen (PSA) expression in LNCaP and 22RV1 cells. Taken together, the data not only demonstrated maspin-mediated repression of AR to augment drug anti-tumor activity but also provided in-depth support for combination of HDAC inhibitors with AR antagonist in CaP therapy.

## Background

Mammary serine protease inhibitor (maspin) is encoded by the SERPIN B5 gene in humans and belongs to the serine protease inhibitor/non-inhibitor superfamily. As it does not undergo the S (stressed) to R (relaxed) conformational transition of active serpins, maspin protein exhibits no serine protease inhibitor activity (Fitzpatrick et al., 1996; Al-Ayyoubi et al., 2004; Law et al., 2005; Narayan and Twining, 2010; Read et al., 2011). Maspin is expressed predominantly in normal mammary epithelial cells but significantly reduced or absent in most of breast carcinomas, prostate cancer (CaP), gastric malignant tissue, and melanoma cancers, yet is overexpressed in pancreatic, gallbladder, colorectal, and thyroid cancers. Thus, maspin may show variant activities and play divergent roles in different cell types. In fact, the expression of maspin mostly appeared to be correlated with better prognosis clinically in prostate, bladder, lung, gastric, colorectal, head and neck, and thyroid cancers and melanoma (Berardi et al., 2013; Kapoor, 2014). Experimentally, maspin showed tumor suppressive activities of blocking cell growth, invasion, and metastasis (Narayan and Twining, 2010; Berardi et al., 2013); inhibiting angiogenesis (Cher et al., 2003); sensitizing tumor cell to drug-induced apoptosis (Liu et al., 2004); and arresting cell cycle progress (Li et al., 2006) in mammary tumors.

In fact, not only the loss of maspin expression was reported to be associated with epithelial malignant transformation and drug resistance in tumor but its allocation of expression in different cellular compartment was also documented with a discrepancy in cancer biology (Lonardo et al., 2006; Martinoli et al., 2014; Reina et al., 2019). Maspin, in addition to extracellular secretion (Dean et al., 2017), was found in the cell membrane, cytoplasm, and nucleus. Further investigation uncovered that the nuclear localization of maspin was correlated with better outcome of cancer therapy in epithelial carcinoma (Lonardo et al., 2006; Machowska et al., 2014), but the mechanism is still uncertain. Our previous studies found that maspin functioned as endogenous histone deacetylase 1 (HDAC1) inhibitor to prevent higher HDAC1 activity-associated epithelial malignant progress, which was through modulating HDAC1-regulated gene expression including up-regulating cytokeratin 18 (CK18), CK8, and glutathione S-transferase PI (GST pi) and down-regulating hypoxia-induced factor 1α (HIF-1α) (Li et al., 2006, 2011; Lee et al., 2012). In this study, we discovered a novel anti-tumor mechanism of maspin to repress androgen receptor (AR) transcription, which augmented the treatment efficacy of AR antagonist enzalutamide in prostate cancer.

## Materials and Methods

### Cell Culture and Reagents

Prostate cancer PC3 and LNCaP cells were purchased from the cell bank of the Chinese Academy of Sciences (Shanghai, China). Prostate cancer 22RV1 cell line was obtained from Nanjing Medical University (Nanjing, China). PC3 cells were maintained in F-12 medium (Gibco/Thermo Fisher Scientific, Shanghai, China) with 10% fetal bovine serum (FBS) (Biological Industries, Israel) supplemented with 1 mmol/l L-glutamine (Sigma-Aldrich, St. Louis, MO, United States) and 1% penicillin/streptomycin (Gibco/Thermo Fisher Scientific, Shanghai, China) according to the manufacturer’s description. LNCaP and 22RV1 were maintained in RPMI 1640 medium with 10% FBS and the same additives as above. All cells were cultured and maintained in a humidified incubator with 6% CO_2_ at 37°C and were tested for free-of-mycoplasma contamination periodically with LookOut Mycoplasma PCR Detection Kit from Sigma-Aldrich (St Louis, MO, United States). DNA methyltransferase inhibitor 5′-Aza-1-(2-deoxy-β-D-ribofuranosyl)cytosine (decitabine, 5′-Aza), and dihydrotestosterone (DHT) were also purchased from Sigma-Aldrich. AR antagonist enzalutamide (Enza), class I HDAC inhibitor entinostat (MS-275), and protease inhibitor MG132 were obtained from Selleck (Houston, TX, United States).

### Recombinant Gene Transfection and Expression of Maspin and AR

The recombinant expression pCDNA3.1 plasmid with SERPIN B5 gene insert in frame and empty vector control were constructed by and purchased from General Biosystems (Anhui, China). Individual plasmid DNA was extracted to transform host *E. coli* strain DH5α cells and the positive clones for maspin expression were selected and used to amplify the plasmid DNA. The purified plasmid DNA were subsequently sequenced and verified. Then, maspin-encoding plasmid DNA was transfected into 22RV1 cells using Lipoteamine 2000 (Invitrogen/Thermo Fisher Scientific, Shanghai, China) followed by western blotting assay for maspin expression. Then, the positive clones were named as M# clones. Control transfection was conducted with the empty vector plasmid DNA and the resulting control cell clones were designated as Neo clones (Li et al., 2011).

Both the recombinant EX-E2325-M02 plasmid with ectopic cytomegalovirus promoter (CMV)-driven AR gene (NM_000044) expression and GFP expression control EX-EGFP-M02 plasmid were obtained from GeneCopoeia (Rockville, MD, United States). Then the individual EX-E2325-M02 plasmid DNA or EX-EGFP-M02 plasmid DNA was stably transfected into CaP PC3 cells respectively as described above. The AR overexpression positive clones were confirmed by western blot and designated as AR# clones. The GFP expression control cell clones were selected and named as Neo clone (Li et al., 2011). The level of maspin, Setd8, and glyceraldehyde-3-phosphate dehydrogenase (GAPDH) was also detected (Supplementary Figure 1).

### Transient Transfection

For maspin knockdown by siRNA, cells cultured in six-well plates were transfected with solution vehicle, a maspin-specific siRNA (GenePharma, Shanghai, China), or scramble oligo control(Scr-siRNA) at 15 mmol/l by using Lipoteamine 2000 (Li et al., 2011). Cells were continuously cultured for another 48 h, and total cell lysate was harvested for protein detection by western blot, or the cells were harvested and total RNA was extracted out for mRNA analysis by quantitative real-time reverse transcript-polymerase chain reaction (qRT-PCR) described later.

### Western Blot Assay

Cells were lysed in radioimmunoprecipitation assay (RIPA) lysis buffer (Nobleryder, Beijing, China) containing 1 mM phenylmethanesulfonyl fluoride (PMSF) and then protein concentration was measured by BCA kit (Thermo Fisher Scientific, Shanghai, China). The protein in cell lysate was denatured by boiling in sample buffer and resolved by running SDS-PAGE. Then the protein in gel was transferred onto a polyvinylidene difluoride membrane (Millipore, Bedford, MA, United States) followed by blocking with 5% milk for 40 min at room temperature (RT). The membrane was incubated with primary antibodies with predetermined dilution overnight at 4°C. Then membrane was washed with Tris-buffered saline with 0.1% Tween-20 and incubated with HRP-conjugated secondary antibodies (Abcam, Cambridge, MA, United States) for 1 h at RT. The expression of protein was detected by enhanced chemiluminescence (ECL) system and autoradiography using Tanon 5500 (Shanghai, China) (Li et al., 2011). The anti-maspin monoclonal antibody (554292) was purchased from BD PharMingen (San Diego, CA, United States), and rabbit antibody against maspin (ab182785), rabbit McAb against AR (ab133273), and β-catenin were purchased from Abcam (Cambridge, MA, United States). Antibodies against p-H2A.X and poly (ADP-ribose) polymerase (PARP) were purchased from Cell Signaling Technology (Boston, MA, United States). The McAb against GAPDH purchased from Good HERE (Hangzhou, China) were used for equal loading and endogenous control. All experiments were conducted independently at least three times and a representative result is presented in the *Results* section.

### Flow Cytometry Assay for Apoptosis

CaP cells (2.5 × 10^5^) were seeded onto six-well plate and treated with enzalutamide (5 μM) and/or MS-275 (1 μM) for 48 h. Then, the cells were harvested and suspended in cold PBS. The cells were then stained with the annexin V-fluorescein isothiocyanate (FITC) and prodidium iodine (PI) kit (BD Biosciences, United States) according to the manufacturer’s instructions followed by flow cytometer sorting (Beckman, CA, United States). The apoptotic cells with annexin V-FITC staining were calculated as apoptotic index (%).

### Isolation of RNA and Quantitative RT-PCR

Total RNA was isolated by using a RiboPure kit (Life Technologies/Thermo Fisher). The cDNA was generated by utilizing High-Capacity cDNA Reverse Transcription Kit (Thermo Fisher Scientific, Shanghai, China) according to the manufacturers’ instruction. Quantitative real-time RT-PCR was performed using POWER SYBR Green Master Mix (Applied Biosystems/Thermo Fisher Scientific) (Li et al., 2011). The following oligonucleotide primers were obtained from GENEWIZ (Suzhou, China): human maspin forward 5′- CTACTTTGT TGGCAAGTGGATGA-3′ and reverse 5′ –ACTGGTTTGGTGT CTGTCTTGTTG-3′; human AR forward 5′- AGCCCCACTGA GGAGACAACC-3′ and reverse 5′-ATCAGGGGCGAAGTAGA GCATC-3′; human PSA forward 5′-GCATCAGGAACAAAAG CGTGA-3′ and reverse 5′-CCTGAGGAATCGATTCTTCAG-3′; human NKX3.1 forward 5′-CAGAGACCGAGCCAGAAAGG-3′ and reverse 5′-CTGAGTGTGGGAGAAGGCAG-3′; and human TMPRSS2 forward 5′- AAACCAGTGTGTCTGCCCAA-3′ and reverse 5′-GCCAGAACCCCAGCTTGTAT-3′. The primer sequence for GAPDH was described previously (Li et al., 2011). PCR reaction started at 50°C for 2 min followed by 95°C for 10 min, 95°C for 15 s and 60°C for 60 s for a total of 40 cycles in ABI 7500 systems (Applied Biosystems/Thermo Fisher Scientific). The expression of individual gene was calculated according to 2^–ΔΔCt^ method after normalizing to the housekeeping gene GAPDH.

### Chromatin Immunoprecipitation Assay

The chromatin immunoprecipitation (ChIP) assay was performed as described previously using SimpleChIP^®^ Enzymatic Chromatin IP (Magnetic Beads) Kit (CST, Boston, MA, United States) according to the manufacturer’s instructions (Li et al., 2011). In brief, CaP LNCaP cells and PC3-AR28 with ectopic AR expression were cultured and cross-linked by 1% formaldehyde. The cells were prepared for chromatin digestion by micrococcal nuclease and sonication according to the instructions. The supernatant containing genomic DNA fragments of 150∼900 bp was collected by centrifugation at 10,000 rpm for 10 min at 4°C and divided into four parts. The first part was used as input control, and the other three parts were incubated with 2 μg anti-maspin antibody, 2 μl normal rabbit immunoglobulin G (IgG, kit provided) as negative control, and 10 μl Rabbit anti-Histone H3 McAb (kit provided) as positive control, respectively. The immunoprecipitated complexes were collected by incubation with protein G magnetic beads. After washing the beads with buffers (low salt and high salt), the cross-linked and pulled down products were reversed by heating the samples at 65°C for 30 min. DNA fragments in the immunoprecipitated complex was purified using spin columns after removing the proteins by adding protease K. Then, the isolated DNA was quantified and qPCR was performed to amplify the individual fragments by using variant primers. A total of eight pairs of specific primer sets (the sequences information are available upon request) were obtained to amplify the variant regional sequence of human AR promoter (refer to TSS and distribute on the range of -3,138∼ + 35). The results from primer 7 was presented. The sequence of primer 7 are the following: forward primer, 5′-ATGCTTTCCTGTTTACAAGTTTGTTCTATACAC-3′; reverse primer, 5′-AGTTACTCTGAATAAAAAGCAGTCTGACAT-3′. The PCR reaction was followed with the following cycling parameters: an activation step of 95°C for 5 min followed by 34 cycles of 95°C for 30 s, 62°C for 30 s, and 72°C for 30 s with a final extension step of 72°C for 5 min. The positive control primer for RPL30 gene amplification was also provided by the kit. PCR products were electrophoresed in 1.5% agarose gel and visualized under ultraviolet light and photographed.

### Microarray Data and Bioinformatics Analysis

The microarray data of maspin and AR gene expression profile from patients with CaP specimens was downloaded from Gene Expression Omnibus (GEO) database^1^ Barrett et al., 2005, 2007). GDS2545 (GSE6919) included 18 normal prostate tissues, 63 normal prostate adjacent to tumor tissues, 65 primary tumor tissues, and 25 metastatic CaP tissues and were analyzed by using Affymetrix Human Genome U95 Version 2 Array (Affymetrix Inc., Santa Clara, CA, United States). GDS4824 (GSE55945) included 8 benign prostate tissues and 13 CaP tissues. GDS1439 (GSE3325) included 4 benign prostate tissues, 5 primary CaP tissues, and 4 metastatic CaP tissues. The above three sets of microarray data were all analyzed using Affymetrix Human Genome U133 Plus 2.0 Array. GDS1390 (GSE2443) included 10 AR-dependent primary CaP tissue and 10 AR-independent primary CaP tissues. GDS4109 (GSE25136) included 79 primary CaP tissues (40 non-recurrent, 39 recurrent). The above two sets of microarray data were analyzed by using Affymetrix Human Genome U133A Array. All the raw array data were pre-processed and analyzed as described (Xu et al., 2018; Mazzini et al., 2019). Log2 conversion and quantile normalization were applied to data if appropriate. Genes with more than 20% missing values were removed. Group differences for gene expression data were analyzed using the Student’s *t* test. The *p* values equal to or below 0.001 were considered significant.

## Results

### Drug-Induced Apoptosis Was a Concomitant Increase of Maspin and Decrease of AR in CaP Cells

**FIGURE 1:**
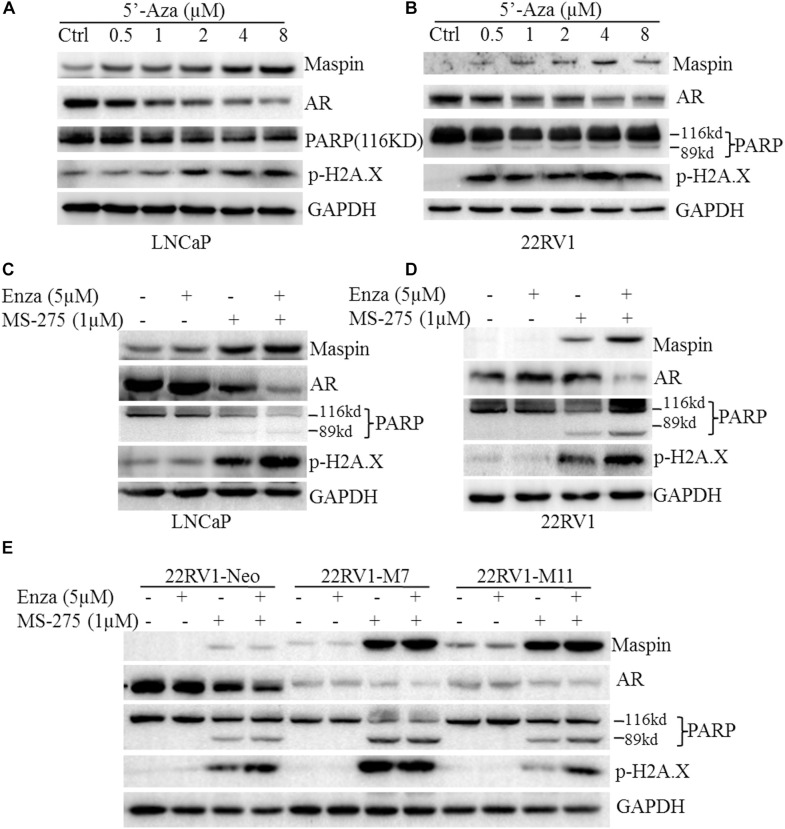
Maspin-mediated downregulation of androgen receptor (AR) was contributed to chemotherapy efficacy. **(A)** Prostate cancer LNCaP cells were treated with 5′-Aza at indicated concentration for 48 h. Then total cell lysates were harvested and western blotting (WB) was conducted to examine the poly (ADP-ribose) polymerase (PARP) cleavage and the level of p-H2AX, maspin, AR, and glyceraldehyde-3-phosphate dehydrogenase (GAPDH). The level of GAPDH was used as equal loading control. **(B)** Prostate cancer 22RV1 cells were treated with 5′-Aza at indicated concentration for 48 h. Then total cell lysate was harvested and WB was conducted to examine the PARP cleavage and the level of p-H2AX, maspin, AR, and GAPDH. The level of GAPDH was used as equal loading control. **(C)** Prostate cancer LNCaP cells were treated with enzalutamide (5 μM) and MS-275 (1 μM) for 48 h. Then total cell lysates were harvested and WB was conducted to examine the PARP cleavage and the level of p-H2AX, maspin, AR, and GAPDH. The level of GAPDH was used as equal loading control. **(D)** Prostate cancer 22RV1 cells were treated with enzalutamide (5 μM) and MS-275 (1 μM) for 48 h. Then total cell lysate was harvested and WB was conducted to examine the PARP cleavage and the level of p-H2AX, maspin, AR, and GAPDH. The level of GAPDH was used as equal loading control. **(E)** The overexpression of maspin clone 22RV1-M7, M11, and the control clone 22RV1-Neo cells were treated with MS-275 (1 μM) and AR antagonist enzalutamide (5 μM) for 48 h. Then total cell lysates were harvested and WB was conducted to examine the PARP cleavage and the level of p-H2AX, maspin, AR, and GAPDH. The level of GAPDH was used as equal loading control.

In general, PARP cleavage and p-H2A.X expression were the classic biomarkers for apoptotic cell death and cellular DNA damage. To investigate its cytotoxicity and therapeutic effect on CaP, demethylation agent 5′-Aza was applied to treat LNCaP and 22RV1 cells at the indicated dose, respectively, and PARP cleavage and p-H2A.X were tested by western blot (WB) analysis. The results showed that treatment with 5′-Aza induced both the PARP cleavage with an 89-kd fragment expression and p-H2A.X level in a dose-dependent manner (Figures 1A,B). Meanwhile, maspin expression was increased but AR expression was downregulated concomitantly along with the increase of 5′-Aza concentration.

It was well documented that the AR antagonist enzalutamide or HDAC inhibitor MS-275 possessed anti-tumor activity. To investigate the synergistic effect of AR antagonist enzalutamide and MS-275 on apoptosis induction, LNCaP or 22RV1 cells were treated with enzalutamide (5 μM) and/or MS-275 (1 μM) followed by WB analysis. The results showed that treatment with MS-275, but not enzalutamide, induced PARP cleavage and p-H2A.X. Meanwhile, the treatment also increased maspin expression but inversely decreased AR expression (Figures 1C,D). Importantly, the combination treatment of enzalutamide with MS-275 showed synergistic effects on the increase of PARP cleavage, p-H2A.X level, and maspin expression and on the decrease of AR level (Figures 1C,D). In addition, this synergistic effects of MS-275 and enzalutamide on CaP cell apoptosis were also confirmed by staining with annexin V-FTIC and PI followed by flow cytometry assay (*p* < 0.001, Supplementary Figure 2).

To further evaluate the role of maspin in sensitizing drug-induced apoptosis in CaP, the maspin-expressed 22RV1-M7, -M11 cells, and the control Neo cells were treated with enzalutamide (5 μM) and/or MS-275 (1 μM) followed by WB analysis. The results showed that, in addition to showing the similar pattern of inverse expression of maspin with AR, M7 and M11 cells showed higher level of both PARP cleavage and p-H2A.X compared with Neo cells while treated with enzalutamide and/or MS-275. Taken together, the data suggested that the above drug-induced CaP cell apoptosis concomitantly increased maspin-associated downregulation of AR.

### Drug-Induced Downregulation of AR Was Not Through Proteasome-Mediated Protein Degradation

**FIGURE 2:**
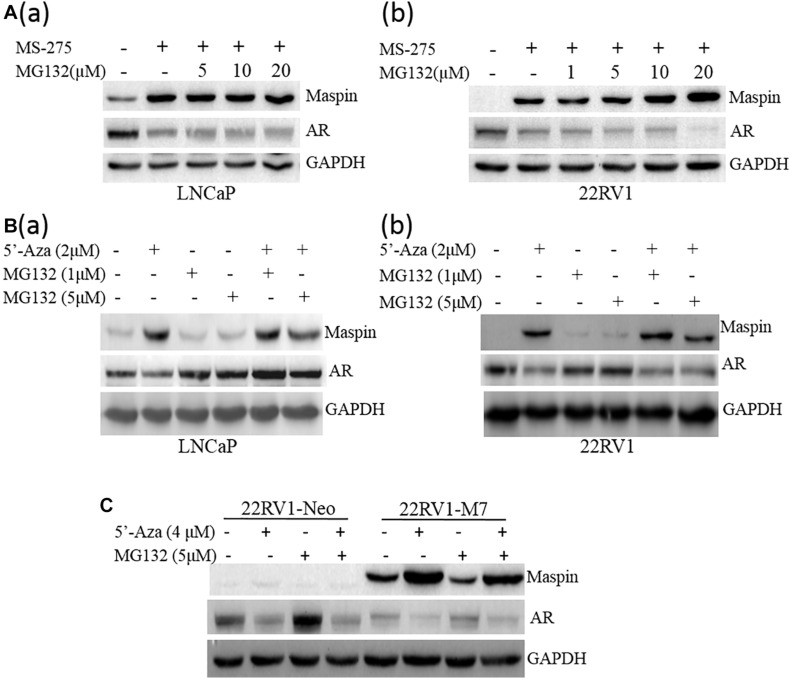
Proteasome inhibition did not rescue the decrease of AR expression. **(A)** Prostate cancer LNCaP (a) or 22RV1 **(b)** cells were treated with class I HDAC inhibitor MS-275 (1 μM) and/or protease inhibitor MG132 at indicated concentration for 48 h. Then total cell lysates were harvested and the expression of AR, maspin, and GAPDH was detected by WB. The level of GAPDH was used as equal loading control. **(B)** Prostate cancer LNCaP **(a)** or 22RV1 **(b)** cells were treated with demethylation agent 5′-Aza and/or protease inhibitor MG132 at indicated concentration for 48 h. Then total cell lysates were harvested and the expression of AR, maspin, and GAPDH was detected by WB. The level of GAPDH was used as equal loading control. **(C)** Overexpression of maspin clone 22RV1-M7 cells and the control clone 22RV1-Neo cells was treated with 5′-Aza and/or MG132 at indicated concentration for 48 h, respectively. Then total cell lysates were harvested and western blot was utilized to examine the expression of AR, maspin, and GAPDH. The level of GAPDH was used as equal loading control.

To exclude the protein instability of AR induced by above drug treatment, LNCaP cells were treated with MS-275 (1 μM) or 5′-Aza with or without the presence of protease inhibitor MG132 followed by WB assay for AR and maspin levels. The results showed that MS-275 indeed induced maspin expression along with the decrease of AR in a dose-dependent manner, and MG132 did not rescue the loss of AR in both LNCaP and 22RV1 cells (Figures 2A (a,b)). A similar phenomena of downregulation of AR was also observed when LNCaP and 22RV1 cells were treated with 5′-Aza (Figures 2B (a,b)). However, treatment with MG132 did rescue the loss of AR including spontaneously occurred or induced by 5′-Aza in LNCaP cells only (Figure 2B (a)). In addition, the above maspin-mediated downregulation of AR was also confirmed in 22RV1-M7 and control 22RV1-Neo cells treated with 5′-Aza and/or MG132 (Figure 2C). Noticeably, AR level was increased in 22RV1-Neo while treated with MG-132. Taken together, the data showed that drug-induced AR downregulation was not through proteasome-mediated protein degradation while it was accompanied with increase of maspin expression.

### Maspin Expression Was Inversely Correlated With AR Level and Its Transcriptional Activity in CaP Cells

**FIGURE 3:**
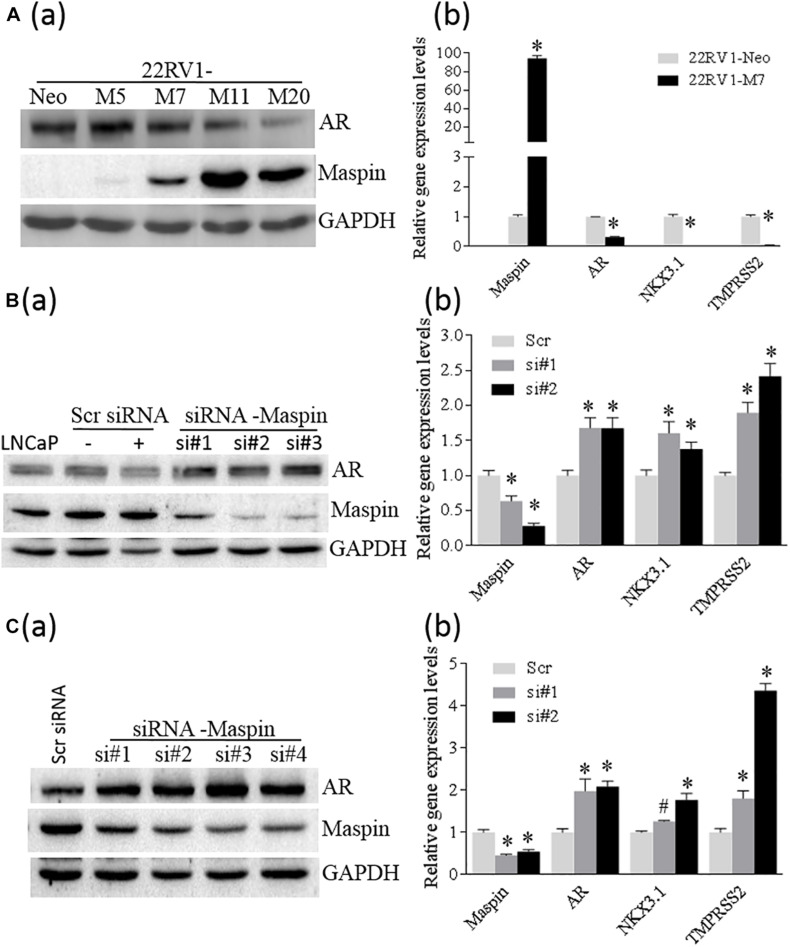
Maspin repressed the expression of AR. **(A)** Overexpression of maspin in prostate cancer 22RV1 cell was conducted by ectopic recombinant maspin gene transfection as described in the section of *Materials and Methods*. The cells transfected with empty vector was used as Neo control. Then western blot was utilized to exam the expression of maspin, AR, and internal loading control of GAPDH in clone cells **(a)**. Meanwhile, real-time PCR was used to evaluate the relative mRNA level of maspin, AR, NKX3.1, and TMPRSS2. The data are presented as an average of three repeats **(b)**. **(B)** Prostate cancer LNCaP cells were transiently transfected with maspin siRNA as described in the section of *Materials and Methods*. The cells transfected with scramble RNA or solution vehicle were used as controls. Then the expression of maspin, AR, and internal loading control of GAPDH in clone cells was analyzed by WB. Three of maspin downregulated clones were designated as si#1, si#2, and si#3 **(a)**. Meanwhile, qRT-PCR was used to evaluate the relative mRNA level of maspin, AR, NKX3.1, and TMPRSS2 in si#1, si#2, and Scr control after normalization with internal GAPDH mRNA. The data are presented as an average of three repeats **(b)**. **(C)** The engineered PC3-AR18 cell clone with AR overexpression was established as described in the section of *Materials and Methods*. Then PC3-AR18 was transiently transfected with maspin siRNA as also described in the section of *Materials and Methods*. The cells transfected with scramble RNA was used as a control. Then the expression of maspin, AR, and internal loading control of GAPDH in clones was analyzed by WB. Four of maspin downregulated clones were designated as si#1, si#2, si#3, and si#4 **(a)**. Meanwhile, real-time PCR was used to evaluate the relative mRNA level of maspin, AR, NKX3.1, and TMPRSS2 in si#1, si#2, and Scr control after normalization with internal GAPDH mRNA. The data are presented as an average of three repeats **(b)**. The bars represent the SE The *p* values were obtained by one-tailed matched pair Student’s *t* tests (*compared with Neo group, *p* < 0.001).

To study whether the expression of maspin in CaP affects AR level, SERPIN 5 gene was transfected into 22RV1 cells through recombinant gene expression technique, and a few positive cell clones were selected and analyzed by WB assay for maspin and AR expression. The results showed that multiple M cell clones with variant levels of maspin expression concomitantly reduced AR level compared with Neo control (Figure 3A (a)). Results from qRT-PCR analysis also showed that the mRNAs of both AR and its regulated downstream NKX3.1 and TMPRSS2 genes were decreased in maspin-expressed M7 clone cells compared with Neo cells (Figure 3A (b)). Furthermore, maspin-specific siRNA was transiently transfected into LNCaP cells and three clones si#1, si#2, and si#3 with maspin knockdown were established. The results from WB analysis showed that, compared with scramble siRNA-transfected clones and the parental LNCaP cells, the levels of AR in si#1, si#2, and si#3 were upregulated (Figure 3B (a)). Accordingly, the mRNA level of AR, NKX3.1, and TMPRSS2 in two siRNA clones si#1 and si#2 was also increased (Figure 3B (b)).

To clarify the role of maspin on AR expression, AR was ectopically overexpressed in PC3 cells through recombinant gene expression technology, and multiple clones with AR expression were identified by WB (Supplementary Figure 1). One of the PC3-AR18 clones with AR expression was selected and then transiently transfected with maspin-specific siRNA. The results from WB assay showed that multiple cell clones of si#1∼si#4 with maspin knocked down expressed increased AR level accordingly (Figure 3C (a)). Similarly, analyzed by qRT-PCR, the mRNA levels of AR, NKX3.1, and TMPRSS2 in maspin knocked down clone of si#1 and si#2 were also increased (Figure 3C (b)). Taken together, the data demonstrated that the overexpression of maspin in CaP cells decreased the AR level and inhibited its transcriptional activity.

### Maspin-Mediated Transcriptional Repression of AR Expression Was Validated by Bioinformatics Analysis and ChIP Assay

**FIGURE 4:**
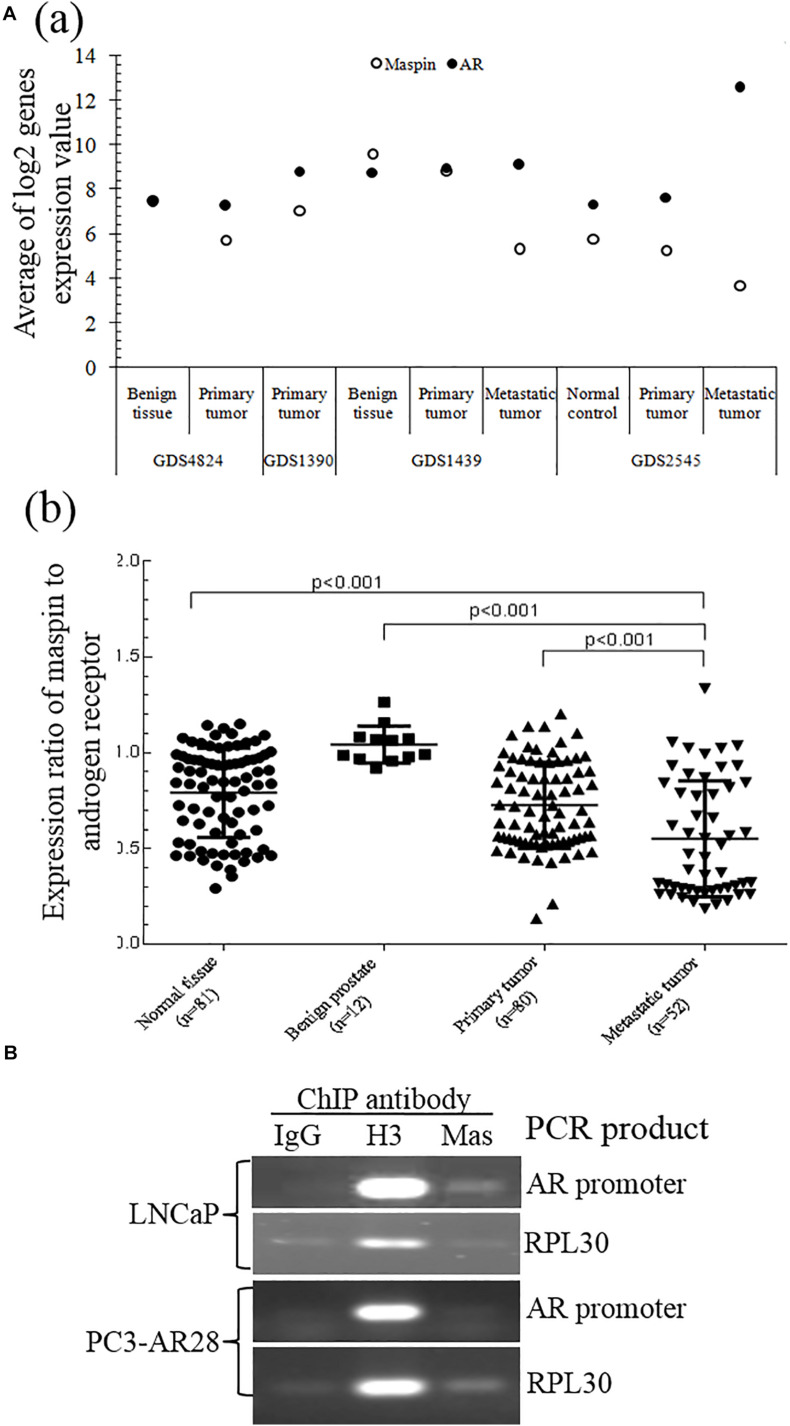
Validation of maspin-repressed AR expression by microarray data from NCBI GEO database and ChIP assay. **(A)** The microarray data of paired expression of maspin mRNA and AR mRNA from clinical specimens were downloaded and extracted from NCBI GEO database (https://www.ncbi.nlm.nih.gov/geo/). The data are presented as an average of Log2 conversion of individual expression values for each study **(a)** and as the group expression ratio of maspin to AR **(b)**. Group differences for gene expression data were analyzed using the Student’s *t* test. The *p* values equal to or below 0.001 were considered as significant difference. **(B)** Prostate cancer LNCaP cells and AR overexpression clone PC3-AR28 cells were cultured and the protein/nuclear acid was cross-linked by adding 1% formaldehyde. The total cell lysate was generated and chromatin immunoprecipitation assay was conducted by adding anti-maspin McAb, anti-H3 antibody (as positive control), or isotype IgG (as negative control). The PCR was performed to amplify the AR promoter regional sequence and reference control RPL30 sequence existing in the set of immunoprecipitation complex.

A study of the National Center for Biotechnology Information (NCBI) GEO data generated from clinical patient tissue specimens of normal prostate, benign prostate, and primary and metastatic CaP also found that there was a differential expression between maspin and AR. Also, downregulation of maspin expression in CaP, which is more significant in metastatic tumors of GDS1439 and GDS2545 groups than in primary and benign or normal tissues, was correlated with upregulation of AR mRNA level (Figure 4A (a)). The expression ratio of maspin to AR was significantly decreased along with the disease progression from benign to metastasis status (Figure 4A (b), *p* < 0.001).

To study the regulation mechanism of maspin on AR transcription and expression, ChIP assay was performed to examine the presence of maspin in the transcriptional regulation complex attached to AR gene promoter. The results showed that the PCR products of positive control RPL30 gene amplified from the IP complex of the IgG negative control, anti-histone 3 antibody positive control, and anti-maspin antibody were consistent in both LNCaP and PC3-AR28 cells. Noticeably, the PCR product of AR promoter fragment amplified by using the AR primer 7 (covers the portion of AR promoter nucleotide 2977∼3177) rather than other AR primers was observed in the anti-maspin antibody-immunoprecipitated complex derived only from LNCaP cell lysate, but not from PC3-AR28 cell lysate. The negative control and positive control were also verified in both cells when AR promoter fragment was amplified using the primer 7 (Figure 4B). These data demonstrated that maspin attached to a certain region of AR promoter in LNCaP cells, rather than in engineered PC3-AR28 cells with artificial CMV promoter-driven AR expression.

Taken together, the evidence from both ChIP assay and GEO demonstrated that maspin indeed repressed AR expression at the transcription level.

### MS-275-Induced Maspin-Mediated AR Repression Augmented the Treatment Effects of Enzalutamide in CaP Cells

**FIGURE 5:**
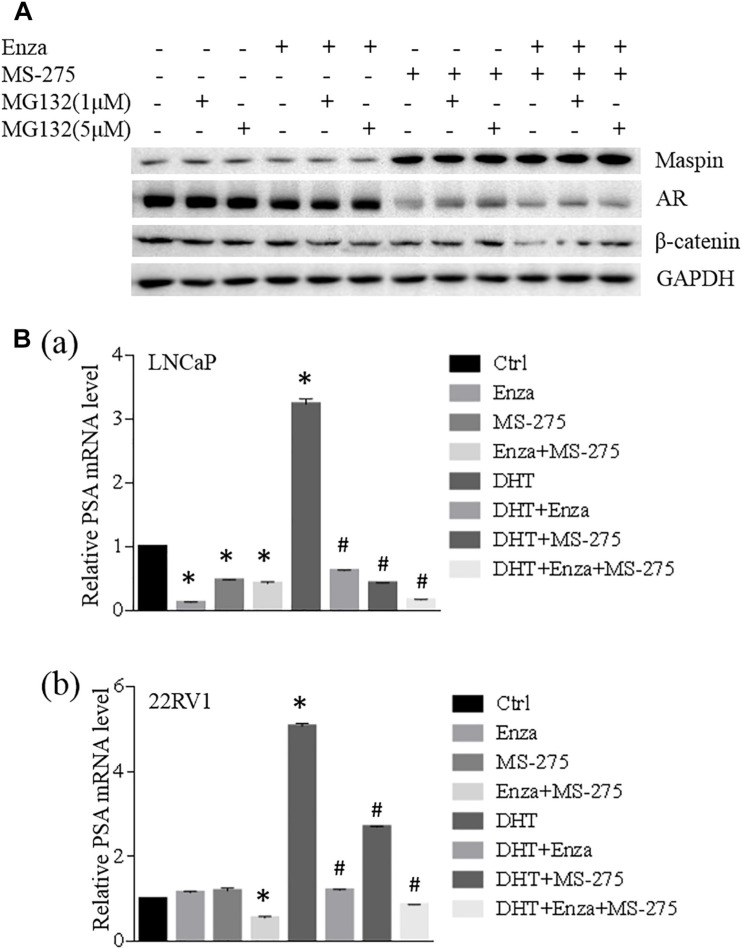
Maspin-mediated AR repression was involved in MS-275-sensitized prostate cancer cells to enzalutamide therapy. **(A)** Prostate cancer LNCaP cells were treated with enzalutamide (5 μM), MS-275 (1 μM), and indicated dose of MG132 for 48 h. Then the cell proteins were extracted by RIPA lysis buffer and the levels of maspin, AR, and β-catenin were evaluated by WB. The level of GAPDH was used as equal loading control. **(B)** Prostate cancer LNCaP **(a)** and 22RV1 **(b)** cells were treated with DHT (10 μM), enzalutamide (5 μM), and MS-275 (1 μM) for 24 h. Total cellular RNA was extracted and the expression of PSA and GAPDH was analyzed by qRT-PCR. The relative expression of PSA gene was calculated after normalization with internal control GAPDH. The experiment was repeated for at least three times and the data are presented as average of three repeats (mean ± SD, *Compared with no treatment control, *p* < 0.001. #compared with DHT treatment group, *p* < 0.001).

To investigate the synergistic effect of MS-275 with AR antagonist enzalutamide on the treatment of CaP cells, LNCaP cells were treated with enzalutamide (5 μM), MS-275 (1 μM), and indicated dose of MG132 for 48 h followed by WB analysis. The results showed that the expression of maspin, AR, and β-catenin had no change after treatment with enzalutamide and/or MG132. Treatment with MS-275 alone induced maspin expression along with downregulation of AR expression, which was rescued partially by addition of MG132. This MG132-mediated partial rescue of AR was diminished by the addition of enzalutamide (Figure 5A). Noticeably, the combination treatment with MS-275 and enzalutamide reduced AR level significantly, and the expression of β-catenin was decreased consistently with AR level (Figure 5A).

When LNCaP or 22RV1 cells were treated with an agonist of the AR DHT alone or in the presence of enzalutamide and/or MS-275 for 24 h followed by qRT-PCR analysis for prostate-specific antigen (PSA) expression, the results showed that treatment with enzalutamide or MS-275 reduced PSA transcription in LNCaP but not in 22RV1 cells; however, the combination treatment with enzalutamide and MS-275 did reduce PSA significantly in 22RV1 cells but has no synergistic effect on LNCaP cells. Interestingly, both MS-275 and enzalutamide alone significantly inhibited DHT-induced PSA expression. The combination treatment with MS-275 and enzalutamide showed additive or synergistic effect on reduction of DHT-induced PSA expression in both LNCaP and 22RV1 cells (Figure 5B). These data indicated that combination treatment of enzalutamide with MS-275 augmented the treatment effect of enzalutamide on PSA production in prostate cancer.

## Discussion

The human AR gene is located on the X chromosome at Xq11-12 (Chang et al., 1988; Trapman et al., 1988), and its expression was reported to be regulated by many mechanisms and factors including AR genomic stability, DNA repair (Mills, 2014), gene transcriptional amplification (Shiota et al., 2011; Jernberg et al., 2017; Zhou et al., 2020), and/or epigenetic modification (Gao and Alumkal, 2010; Katsogiannou et al., 2015; Liao and Xu, 2019). Variant transcription factors and co-factors were reported to regulate AR expression (Imamura, 2011; Wang et al., 2016). Typically, AR gene transcription was reported to be regulated by age-dependent transcription factor (ADF), Sp1, serum response factor (SRF), NF-kappa B p50/p50 homodimer, and possibly AP3 (Song et al., 1995; Supakar and Roy, 1996).

Tumor suppressive maspin was reported to sensitize tumor cells to drug-induced apoptosis (Liu et al., 2004; Lee et al., 2012; Berardi et al., 2013). In the current study, apoptosis induction by drug treatment resulted in not only an increase of maspin expression but also downregulation of AR concomitantly in CaP cells. Then, modulation of maspin expression by gene recombinant or siRNA technology also showed the inverse correlation expression of maspin with AR and consequently the AR-regulated downstream gene transcription. Treatment with protease inhibitor MG132 did not rescue maspin-mediated downregulation of AR. Furthermore, ChIP assay using anti-maspin antibody also identified that maspin was bound with a portion of AR promoter sequence and co-existed in the immunoprecipitated complex. Taken together, the data indicated that maspin was involved in AR transcription repression in CaP.

It was also well known that abnormal and persistent activations of AR-mediated signaling pathway were the major mechanism of CaP development and castration-resistant prostate cancer (CRPC) formation (Mills, 2014; Katsogiannou et al., 2015; Gritsina et al., 2019). Thus, as a tumor-suppressive molecule, maspin’s anti-tumor activity was further explored to be also through, at least in part, repressing AR expression. It was noticed that maspin–AR promoter interaction was observed in LNCaP cells rather than in recombinant engineered PC3-AR28 cells which contained an artificial CMV transcriptional promoter to derive AR expression (Figure 4B), and downregulation of maspin by siRNA enhanced AR level in PC3 clones with artificial ectopic recombinant AR expression (Figure 1C). Hence, we proposed that maspin was recruited by AR-specific transcription factor as a co-regulator (e.g., endogenous HDAC 1 inhibitor) to join the transcriptional complex and to mediate AR transcription repression.

HDAC 1 was reported previously to be involved in the regulation of AR gene transcription, and the outcomes of its mediated AR activity was reported controversially (Gaughan et al., 2002; Rokhlin et al., 2006; Park et al., 2018). Gaughan et al. reported that HDAC 1 along with other transcriptional factors (TFs) was recruited to AR promoter and formed a co-repressive complex to inhibit AR gene transcription (Gaughan et al., 2002). However, Rokhlin et al. reported that restraining HDAC 1 activity by trichostatin A inhibited AR gene expression and induced cell death in AR-positive prostate cancer (Rokhlin et al., 2006). More recently, treatment with HDAC inhibitors (vorinostat or CG200745) decreased the levels of full-length AR (AR-FL), AR splice variants (AR-Vs), PSA, and anti-apoptotic Bcl-2 (Park et al., 2018). Indeed, HDAC-mediated gene transcription repression was evidenced. Meanwhile, HDAC-mediated gene transcription activation and HDAC inhibitor-induced transcription repression were also well illustrated previously (Kim et al., 2013; Greer et al., 2015). It was reported that TF–chromatin interactions were highly dynamic which may trigger significantly divergent outcomes of gene transcription along with co-regulators (Shang et al., 2002; Tesikova et al., 2016). For instance, both the co-activators of GRIP1 and CBP and the co-repressors of NCoR, SMRT, HDAC1, and HDAC2 could be recruited by AR to PSA gene promoter, but the PSA expression was eventually determined either by co-activator complex formation initiated by the involvement of coordination between both the promoter and enhancer of PSA gene or by co-repressor complex formation initiated by the involvement of promoter only (Shang et al., 2002). In the current study, the expression of endogenous HDAC 1 inhibitor maspin or treatment with class I HDAC inhibitor MS-275 showed consistently to decrease AR expression (Figures 3A (a), 1C–E, 5A). Thus, it could be speculated that, as an endogenous HDAC1 inhibitor, this maspin-mediated AR gene transcription repression was through, at least in part, inhibiting HDAC1-mediated gene transcription activation (Rokhlin et al., 2006; Park et al., 2018). However, the precise mechanism of maspin-mediated AR transcription repression (e.g., the recruiter of TF, etc.) still remains to be further addressed. It was reported that AR could mediate the loss of maspin expression through recognition of negative HRE element (Zhang et al., 1997; He et al., 2005), and androgen ablation increased the expression of maspin (Zou et al., 2002). However, the data from our study of drug-modulated AR activity (Figures 1C–E, 2C, 5A) and recombinant AR gene expression (Supplementary Figure 1) did not show AR regulative activity on maspin expression. The inconsistency may be due to using different cell lines and disparate treatment strategy in our study, as AR-mediated maspin transcription repression was tissue and cell specific (Zhang et al., 1997).

The drugs enzalutamide, MS-275, 5′-Aza, and MG132 showed their potent anti-tumor activity of inducing DNA damage and promoting apoptosis, but translational implementation of these agents into clinical practice actually faced variant challenges. For example, enzalutamide was commonly used to treat CaP but is only effective for a certain period of time of disease followed by drug-induced resistance. Thus, looking for a long-term and effective treatment strategy for CaP is desirable. In this study, the class I HDAC inhibitor MS-275 was found to not only promote the therapeutic effect of enzalutamide in the drug-sensitive LNCaP cell (Figures 1C, 5B (a)) but also sensitize the drug-resistant 22RV1 cells to enzalutamide treatment (Figures 1D, 5B (b)). Consequently, gathering our and others recent data (Hu et al., 2019; Tang et al., 2019), we proposed that HDAC inhibitor could be useful in clinic cancer chemotherapy if an appropriate strategy was designed. In addition, AR was demonstrated to be a novel target of maspin which may reflect an additional characteristic of maspin for its anti-tumor activity and enriched its anti-tumor potency.

As one of CaP master regulators, AR expression and its activity dominate the disease development and progression. Currently, androgen deprivation therapy (ADT) remains the foundation but not the cure of treatment for CaP. However, after initial regression, the disease comes back in a more progressive form of CRPC with molecular alterations of AR. The second generation of AR pathway inhibitors, such as enzalutamide and abiraterone, were commonly used for high-risk locally or systemically advanced CaP (Borgmann et al., 2018; Hussain et al., 2018; Alpajaro et al., 2019; Mateo et al., 2019). Thus, targeting AR with more specific and higher affinity compound to block AR-mediated downstream signaling becomes desirable for therapeutic improvement on prostate oncology (Dalal et al., 2018; Weissenrieder et al., 2019). Therefore, utilizing the characteristic of maspin-mediated AR repression appropriately could open a new avenue for AR-related CaP management.

## Conclusion

Taken together, these data not only demonstrated maspin-mediated repression of AR expression to augment epi-drug’s anti-tumor activity but also provided in-depth support for combination treatment strategy with AR antagonist and HDAC inhibitors in CaP therapy.

## Data Availability Statement

The datasets presented in this study can be found in online repositories. The names of the repository/repositories and accession number(s) can be found in the article/ Supplementary Material.

## Author Contributions

ST and XuL generated most of the data. JJ generated data from bioinformatics analysis. HC, JG, and CH helped to generate the western blot and RT-PCR data. HM helped to design the experiment and discussed the data. XiL designed the project, directed the research, evaluated the data, and finalized the manuscript. All authors participated in data discussion, read and approved the final manuscript.

## Conflict of Interest

The authors declare that the research was conducted in the absence of any commercial or financial relationships that could be construed as a potential conflict of interest.

## Footnotes

1 https://www.ncbi.nlm.nih.gov/geo/

## Supplementary Material

Supplementary Figure 1 Evaluating the effect of AR overexpression on maspin level. Prostate cancer PC3 cells were stably transfected with AR gene as described in the section of *Materials and Methods*. The multiple AR positive clones and control Neo clone were selected and cultured in growth medium. Total cell lysates were harvested and analyzed for the expression of AR, maspin, Setd8, and GAPDH by western blot. The level of GAPDH was used as equal loading control, and the expression of Setd8 served as additional non-specific molecule control.

Supplementary Figure 2 Flow cytometry assay for Apoptotic cell death. LNCaP **(A)** or 22RV1 **(B)** cells (2.5 × 10^5^) were seeded into six-well plate and treated with enzalutamide (5 μM) and/or MS-275 (1 μM). Then the cells were harvested and were stained with annexin V-FITC and PI kit followed by flow cytometer sorting (Beckman, United States). The apoptotic cells with annexin V-FITC staining were calculated as apoptotic index (%). Three independent experiments were conducted, and a representative result was presented **(a)** and statistically significant data was also presented **(b)**. ^*^*p* < 0.001, indicated significant difference compared with control, MS-275, or enzalutamide alone groups.

## Abbreviations

ADT: androgen deprivation therapy
AR: androgen receptor
CRPC: castration-resistant prostate cancer
HDAC: histone deacetylase
maspin: mammary serine protease inhibitor
PSA: prostate-specific antigen
TF: transcriptional factor.
