# Supplementary figures and images for: Tumor Suppressive Maspin-Sensitized Prostate Cancer to Drug Treatment Through Negative Regulating Androgen Receptor Expression
Source: Front Cell Dev Biol. 2020 Oct 26;8:573820. doi: 10.3389/fcell.2020.573820 (PMC7649228; doi:10.3389/fcell.2020.573820)

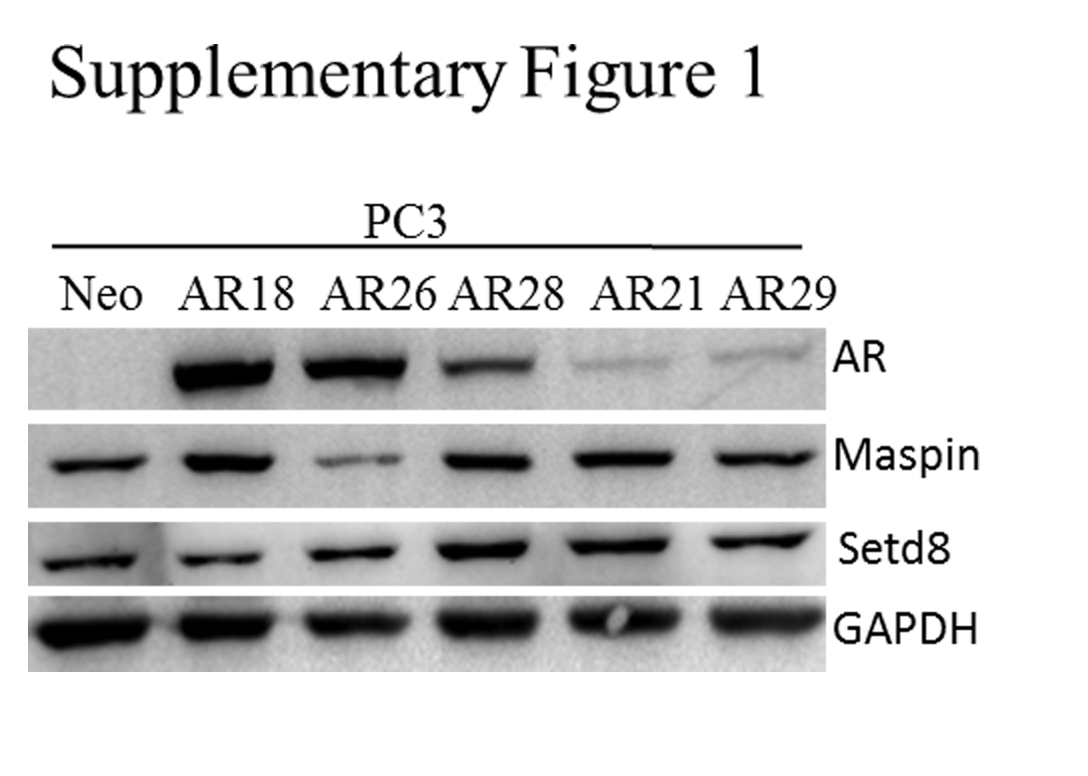

Supplement: Supplementary Figure 1 — Evaluating the effect of AR overexpression on maspin level. Prostate cancer PC3 cells were stably transfected with AR gene as described in the section of Materials and Methods. The multiple AR positive clones and control Neo clone were selected and cultured in growth medium. Total cell lysates were harvested and analyzed for the expression of AR, maspin, Setd8, and GAPDH by western blot. The level of GAPDH was used as equal loading control, and the expression of Setd8 served as additional non-specific molecule control. [file Image_1.TIF]

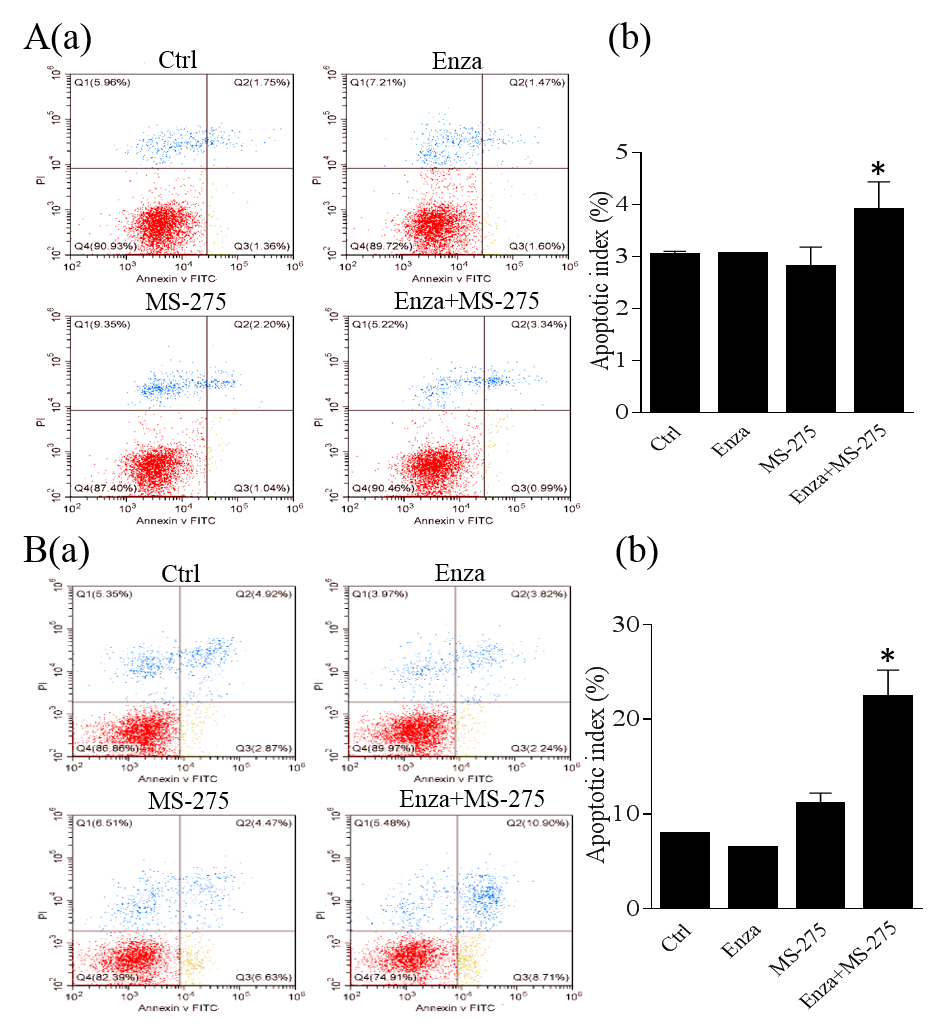

Supplement: Supplementary Figure 2 — Flow cytometry assay for Apoptotic cell death. LNCaP (A) or 22RV1 (B) cells (2.5 × 10^5^) were seeded into six-well plate and treated with enzalutamide (5 μM) and/or MS-275 (1 μM). Then the cells were harvested and were stained with annexin V-FITC and PI kit followed by flow cytometer sorting (Beckman, United States). The apoptotic cells with annexin V-FITC staining were calculated as apoptotic index (%). Three independent experiments were conducted, and a representative result was presented (a) and statistically significant data was also presented (b). *p < 0.001, indicated significant difference compared with control, MS-275, or enzalutamide alone groups. [file Image_2.TIF]
